# CVON: a laudable strategy waiting for societal impact

**DOI:** 10.1007/s12471-015-0672-y

**Published:** 2015-03-04

**Authors:** E.E. van der Wall

**Affiliations:** Netherlands Society of Cardiology/ Holland Heart House, Moreelsepark 1, 3511 EP Utrecht, The Netherlands

In the year 2010, The Netherlands Cardiovascular Research Committee (CVON, www.cvon.eu) was installed based on an initiative of The Netherlands Heart Foundation (currently heart foundation). At that time, funding of research by the Heart Foundation changed from a bottom-up approach to a top-down approach. The Heart Foundation was assisted by the following organisations: The Netherlands Federation of University Medical Centers (NFU), the Royal Netherlands Academy of Arts and Sciences (KNAW) and The Netherlands Organisation for Scientific Research (NWO/ZonMw).

In the spring of 2011, a call for nationwide proposals went out for the first time. Consequently, large, nationwide consortia were formed with the task to formulate integrated research proposals. All proposals were carefully reviewed by a dedicated International Scientific Advisory Council (ISAC) which decided in January 2102 to fund three programmes for a total of 3.0–5.0 million € each. The following three large programmes were granted: a programme on regenerative medicine (HUSTCARE), one searching for new biomarkers and therapeutic targets for atherosclerosis (GENIUS), and finally a programme on the development of drugs for heart failure (ARENA).

Likewise, in 2012, the same policy was followed and a total of four programmes were supported in 2013: (1) 1VALVE: One Valve for Life; (2) HBC: The Heart-Brain Connection: The Missing Link in the Pathophysiology of Vascular Cognitive Impairment; (3) PHAEDRA: Pulmonary Hypertension and Associated Right Heart Failure: Breaking the Vicious Circle; and (4) PREDICT: Predict Sudden Cardiac Death—Uncover Novel Genes and Mechanisms and Test Hypotheses in the Clinic.

The main benefit of the CVON approach is bringing researchers in the same field together (from experimental to clinical) potentially leading to a better focus and improved reorientation in order to perform joint research at a higher level. The benefits of this synergetic strategy were also recognised outside The Netherlands [1]. However, in 2014 it was felt that a potential drawback of the large programmes was that only a limited number of programmes could be supported, mainly directed by older established investigators, while CVON should also serve and stimulate young researchers [2].

To that purpose, the Heart Foundation found an adequate solution by funding smaller programmes for 1.0–1.5 million € each. As a result, in January 2015 the following six programmes were supported by ISAC, consisting of three large programmes: (1) ENERGISE (metabolic activation to prevent cardiovascular disease), (2) RACE V (prevention of complications in atrial fibrillation), (3) RE-CONNECT (diastolic heart failure in diminished kidney function), and three smaller programmes: (1) CONCOR-GENES (prediction of complications in congenital heart disease), (2) REMAIN (more effective treatment after myocardial infarction) and (3) DOSIS (new causative mechanisms in hereditary cardiovascular diseases). In total, the Heart Foundation invested 19.5 million € in these six programmes. For an extensive description of the supported programmes see www.hartstichting.nl.

Altogether, the granted CVON programmes nicely fit within the recently determined research agenda of the Heart Foundation consisting of five themes: (1) early recognition of cardiovascular disease, (2) cardiovascular disease in women, (3) improved treatment of heart failure and atrial fibrillation [3], (4) acute treatment of cerebrovascular accidents and (5) new ways of continuing a healthy lifestyle [4].

The Heart Foundation should be credited for taking the initiative of starting CVON and, more importantly, for having continued this approach for several years. Many millions of Euros have been invested in scientific research with the aim to unite different research groups across the country both at the clinical and basic level. However, the final goal of the CVON programmes should stretch much further than just the formation of consortia. CVON should encourage young researchers, stimulate efforts in performing joint research and pay attention that the results are published in high-quality journals. But foremost, the scientific results have to be translated into benefits for the community. Ultimately, it is the community that raised the money, making all these scientific efforts possible. Consequently, societal impact is ‘the heart of the matter’. Of course, this is an issue which can only be assessed in the long run. Nevertheless it should be the ‘red wire’ in performing scientific research today. The time of ‘l’art pour l‘art’ in science has gone as was also emphasised recently by a group of Dutch scientists called Science in Transition [5].

Fortunately, the Heart Foundation-directed CVON programmes yield sufficient confidence that they will serve the community both in prevention and treatment of cardiovascular disease.
